# Longitudinal Relationships of Physical Activity, Sedentary Time, Cardiorespiratory Fitness, and Muscular Fitness with Body Fatness in Preschoolers

**DOI:** 10.3390/sports12090237

**Published:** 2024-08-30

**Authors:** Kirkke Reisberg, Eva-Maria Riso, Liina Animägi, Jaak Jürimäe

**Affiliations:** 1 Department of Physiotherapy and Environmental Health, Tartu Health Care College, 50411 Tartu, Estonia; liinaanimagi@nooruse.ee; 2 Institute of Sport Sciences and Physiotherapy, Faculty of Medicine, University of Tartu, 51008 Tartu, Estonia; eva-maria.riso@ut.ee (E.-M.R.); jaak.jurimae@ut.ee (J.J.)

**Keywords:** physical activity, sedentary time, cardiorespiratory fitness, muscular fitness, body fatness, children

## Abstract

There is still insufficient knowledge about the potential benefits of physical activity and fitness or the adverse impact of sedentary behaviours on body composition at preschool age. Therefore, we aimed to study the relationships of moderate-to-vigorous physical activity (MVPA), sedentary time (ST), cardiorespiratory fitness (CRF), and muscular fitness (MF) with body fat percentage (BF%) in boys and girls. Participants were investigated twice: in the final year of kindergarten, when the boys’ median age was 7 years and the girls’ median age was 6 years (*p* = 0.240), and again in the first grade of school. MVPA and ST were measured with an accelerometer, BF% was derived from skinfold thicknesses, CRF was measured with a 20 m shuttle run test, and MF was represented by the mean z-scores from standing long jump and relative upper-limb strength. In girls, higher ST (β = 0.587, *p* = 0.021) and lower MF (β = −0.231, *p* = 0.009) at preschool age were related to higher BF% in the first grade of school after adjustment for confounders. MVPA and CRF in preschool children were unrelated to BF% in school among boys and girls. In conclusion, sitting less and having greater muscular fitness at preschool age appear to be beneficial for lower body fatness in the first grade among girls, but not in boys. MVPA and CRF at preschool age are unrelated to body fatness at school age in boys and girls. Our results indicate that girls may especially benefit from decreasing sedentary behaviours and increasing upper- and lower-limb muscular strength at preschool age for a healthy weight profile in the first grade of school.

## 1. Introduction

The prevalence of overweight (and obesity) among children has dramatically increased globally [1]. Adiposity is related to cardiovascular, endocrinologic, gastrointestinal, orthopaedic, and psychological complications [2], with an increased economic burden [3]. Also, research shows that, in preschool, children spend a very long time engaging in sedentary behaviours; they are sedentary about 90% of the time, and only a small part of the day is spent performing physical activities [4,5]. In addition to the range of health benefits of physical activity (PA) [6], physical fitness (PF) is also linked to health and well-being from an early age [7,8,9]; yet, the overall fitness level of preschool children, among both boys and girls aged five, six, and seven years, is unsatisfactory [10]. It is not well understood whether extensive sedentary time (ST) at preschool age has a detrimental association with later body fatness measures or whether physical activity and fitness have beneficial associations once children have entered primary school. The preschool years are crucial for studying the determinants of childhood obesity, as this is the time when PA habits are becoming established [11].

Research shows that children who do not meet the sedentary screen time guidelines (<1 h/day) in preschool are at higher risk of overweight and obesity [12]. Overall, studies on the associations between ST and adiposity during early childhood demonstrate positive [12,13,14] as well as no [14,15,16,17,18,19,20,21] associations.

Studies on objectively measured MVPA and adiposity in early childhood report either negative [15,16,17,18,20] or no [15,17,19,20,21,22] associations.

In addition to some inconsistency in the findings, longitudinal research [13,19,20,21,22] on the relationships between objectively measured ST and PA levels and body fatness in preschool-aged children is scarce. Only a few cross-sectional [14] and longitudinal [13] studies have investigated preschool-aged boys and girls separately, leaving it unclear whether sedentary behaviour and PA have different impacts on adiposity in boys compared to girls.

Research among preschool children regarding the links between CRF, MF, and BMI shows mostly no associations, since no relationships regarding CRF [23,24,25,26], lower-limb muscular strength [23,24,25], and relative upper-limb muscular strength [26] have been reported. However, a positive association between upper-limb muscular strength [23,25] and BMI exists, while an inverse relationship between CRF [27] and relative lower-limb muscular strength [26] and BMI has been demonstrated. Some of the lacking and contradictory associations between PF and BMI in preschoolers might be attributable to the opposing associations of PF with body leanness and fat mass values [25].

Studies on the associations of CRF and lower-limb muscular strength with BF% and fat mass index have demonstrated inverse [23,25,26,27,28] or no [28] relationships, while upper-limb muscular strength was either positively [26] or negatively [28] related to BF% and fat mass index among preschoolers, or no associations were found [23,24,25,26].

If children begin school with excessive weight, they start their education on a negative trajectory that can be extremely difficult and expensive to reverse. Greater awareness about the associations between ST, PA, PF, and body fatness will support the development of interventions to encourage healthy lifestyles in preschool-aged children. Previous studies have rarely accounted for the gender differences in body fat accumulation and distribution [29,30], time being engaged in physical activities [31], and physical fitness level [10,24] that are already evident at an early age, thus restricting our understanding of whether the findings are generalizable to both genders or whether they are gender-specific. Recognizing gender-specific associations could contribute to the development of more specific intervention strategies for preschoolers aimed at obesity prevention during the period of transition from preschool to school.

This longitudinal study aimed to explore the following: (1) whether moderate-to-vigorous physical activity (MVPA) and sedentary time (ST) during preschool are related to body fatness in the first grade of school among boys and girls; (2) whether cardiorespiratory fitness (CRF) and muscular fitness (MF) in preschool are related to body fatness in the first grade of school among boys and girls. We hypothesized that higher MVPA, CRF, and MF and lower ST in preschool would be related to lower body fatness a year later in school.

## 2. Materials and Methods

### 2.1. Participants

The authors contacted 13 kindergartens in Tartu County, Estonia, inviting children and their parents to join the study. From 400 children, 284 volunteered to participate in the measurements taken in Spring 2016, when children were attending their last year of kindergarten.

Children (*n* = 200) and their parents were assessed again in Spring 2017, when the children were in their first year of school.

Valid baseline data from 77 children (51.9% boys) were included in the analysis. Complete data on variables used in the analyses on the associations of MVPA and ST in preschool with BF% in school were available for 48 boys and 40 girls. Complete data on variables used in the analyses on the associations of CRF and MF in preschool with BF% in school were available for 56 boys/54 girls and 58 boys/55 girls, respectively.

Parents’ educational levels were graded as basic, general secondary/vocational, or higher. The educational levels of higher-educated parents were included.

This study was approved by the Research Ethics Committee of the University of Tartu (references 254/T-13 and 266/T-8) and is consistent with the ethical standards of the Declaration of Helsinki.

### 2.2. Assessment of Body Composition

Body height was measured using a Seca 213 (Hamburg, Germany) portable stadiometer to the nearest 0.1 cm. An A&D Instruments (Abington, UK) digital medical scale was used to measure body weight (to the nearest 0.05 kg). Body weight (kg) divided by body height (m)^2^ gave body mass index (BMI). BMI cut-off values were applied to define children with overweight (17.71 and 17.53 kg/m^2^ for boys and girls, respectively) and obesity (18.16 and 18.03 kg/m^2^ for boys and girls, respectively) at baseline [32]. Skinfold thickness was measured to the nearest 0.2 mm in triplicate with a Holtain caliper (Crymych, UK), including triceps and subscapular, following a standardized protocol [33]. Body fat percentage (BF%) was calculated using the formula developed by Slaughter et al. [34].

### 2.3. Assessment of Physical Activity

The hip-mounted Actigraph GT3X accelerometer (ActiGraph LLC, Pensacola, FL, USA) was used to estimate PA and ST. Children were instructed to wear the accelerometer for seven days and to remove it only when performing water-based activities (e.g., bathing, swimming) and during sleeping [35]. The data were collected in intervals of 15 s. Participants with at least three days (incl. one weekend day) of ≥10 h daily accelerometer wear time were included in the analysis [36]. Non-wearing time was characterized as ≥20 min periods of consecutive readings of zero counts and was excluded from the analysis [37]. The acceleration cut-off points for classifying ST, light PA (LPA), moderate PA (MPA), and vigorous PA (VPA) were as follows: <100, 100 to 1999, 2000 to 3999, and ≥4000 counts per minute, respectively [38,39]. The average time per day spent in each intensity zone was calculated as follows: (weekdays × 5 + weekends × 2)/7 [40]. Moderate-to-vigorous PA (MVPA) was calculated by summing the time spent in MPA and VPA [41,42]. Accelerometer wear time (AWT) was obtained by summing ST, LPA, MPA, and VPA [19].

### 2.4. Assessment of Physical Fitness

CRF was investigated via a 20 m shuttle run test [43] performed once. Children were told to run back and forth over a distance of 20 m marked with two lines. The test commenced at a speed of 8.5 km/h, and the running speed was increased after every minute by 0.5 km/h. The test ended when the children could not follow the set pace of the test. The number of laps completed was recorded [44].

Lower-limb muscular strength was assessed by a standing long jump (SLJ) test (cm). Children stood on a starting line with their legs parallel. They were instructed to jump forward as far as possible. Arm swing was allowed for the jump [45]. The best result of two attempts was taken into account [25].

Upper-limb muscular strength was assessed by a digital dynamometer (Digital TKK 5401, Grip D, Takei, Tokyo, Japan) in a standing position, with the shoulder being tested slightly apart from the body and the elbow extended. Children were asked to squeeze the dynamometer continuously for 2–3 s. They performed two attempts with each hand, and the best result was taken into account. The mean of left and right handgrip strength (kg) was calculated [46] and expressed relative to body mass (in kg/kg) [47].

Muscular fitness z-score was generated from the mean of gender-specific z-scores of relative upper- and lower-limb muscular strength and has been described in previous studies [47].

### 2.5. Statistical Analysis

Statistical analysis was conducted with SPSS software (version 20.0; SPSS, Inc., Chicago, IL, USA). *p* < 0.05 was considered statistically significant. The data from boys and girls were analysed separately. Continuous variables were assessed for normality by the Shapiro–Wilk test. Continuous variables that were normally distributed were compared by Student’s *t*-test, the Mann–Whitney U test was used for skewed variables, and the chi-square test was applied for categorical variables. Multiple linear regression analysis was applied to examine the relationship of MVPA, ST, CRF, and MF z-score in preschool with BF% in school. Baseline values of exposures (MVPA, ST, CRF, MF z-score) and baseline values of the outcome (BF%) [48] were included in the adjusted model. In addition, age [19,39,48,49] and parental education [14,39,48,50] at baseline were included in the adjusted model, and physical activity data were additionally adjusted for baseline AWT [19,49,51,52]. There was no multicollinearity problem, as the values of the variance inflation factor between variables were less than five.

## 3. Results

### 3.1. Characteristics of Children at Baseline

Compared to girls, boys were taller (*p* < 0.001) (127 ± 5.5 vs. 124 ± 6.6 cm), had higher (*p* = 0.010) median weight [26 (interquartile range of 7) vs. 24 (interquartile range of 6) kg], and had a lower BF% (*p* < 0.001) (20.6 ± 4.6 vs. 22.6 ± 5%). Boys had higher median MVPA (*p* = 0.003) in comparison to girls [62.9 (interquartile range of 28.9 vs. 58.5 (interquartile range of 28.3) min/day]. Regarding PF, the handgrip strength of boys was greater (*p* = 0.007) than that of girls [11.5 (interquartile range of 3) vs. 9.9 (interquartile range of 2.7) kg]. The prevalence of boys and girls with overweight/obesity was similar (*p* = 0.170) (17.9 vs. 9.9 %) (Table 1).

Parents’ education level was not different (*p* = 0.765) between boys and girls (basic education 1.4 vs. 3%, general or vocational secondary 15.7 vs. 17.9%, and university degree 82.9 vs. 79.1%, respectively).

### 3.2. Relationships between MVPA, ST in Preschool, and BF% in School

Among boys, MVPA in preschool was unrelated to BF% in school. In the adjusted model (R^2^ = 0.601, *p* < 0.001), BF% in preschool had a positive relationship with BF% in school (*p* < 0.001). In girls, MVPA in preschool was not related to BF% in school. In the adjusted model (R^2^ = 0.703, *p* < 0.001), BF% in preschool was positively related to BF% in school (*p* < 0.001) (Table 2).

Among boys, ST in preschool was not related to BF% in school. In the adjusted model (R^2^ = 0.627, *p* < 0.001), BF% in preschool was positively related to BF% in school (*p* < 0.001). In girls, ST in preschool was not related to BF% in school. In the adjusted model (R^2^ = 0.726, *p* < 0.001), higher ST (*p* = 0.021), higher BF% (*p* < 0.001), and lower AWT (*p* = 0.021) in preschool were related to higher BF% in school (Table 2).

### 3.3. Relationships between CR, Muscular Fitness in Preschool and BF% in School

Among boys, CRF in preschool was not related to BF% in school. In the adjusted model (R^2^ = 0.656, *p* < 0.001), BF% (*p* < 0.001) and age (*p* = 0.027) in preschool were positively related to BF% in school. In girls, CRF in preschool was not related to BF% in school. In the adjusted model (R^2^ = 0.650, *p* < 0.001), BF% in preschool was positively related to BF% in school (*p* < 0.001) (Table 2).

Among boys, higher MF (R^2^ = 0.069, *p* = 0.042) in preschool was related to lower BF% in school. In the adjusted model (R^2^ = 0.641, *p* < 0.001), BF% (*p* < 0.001) in preschool was positively related to BF% in school. In girls, higher MF (R^2^ = 0.122, *p* = 0.006) in preschool was related to lower BF% in school. In the adjusted model (R^2^ = 0.698, *p* < 0.001), lower MF (*p* = 0.009), higher age (*p* = 0.011), and higher BF% (*p* < 0.001) in preschool were related to higher BF% in school (Table 2).

## 4. Discussion

We aimed to investigate whether MVPA, ST, CRF, and MF in preschool age are related to BF% in the first school year among boys and girls. The first main finding was that ST at preschool age was positively associated with body fatness in the first grade of school once controlled for baseline age, BF%, AWT, and parental education in girls, but not in boys. MVPA in preschool was unrelated to body fatness in the first grade of school after adjustment for confounding factors in boys and girls. Secondly, we found that higher MF in preschool was related to lower body fatness in the first grade in girls but not in boys after adjustment for baseline age, BF%, and parents’ educational level.

Research has shown limited and mixed findings on the relationships between ST and body fatness among preschool-aged children [12,13,14,15,16,17,18,19,20,21]. For example, España-Romero et al. [14] reported no associations of ST and BMI z-score with waist circumference in young boys and girls, although higher ST was associated with higher waist circumference among girls at the 90th percentile. However, BMI is not the most accurate method for predicting fat mass in young children [53] since it does not distinguish between fat mass and lean mass [54]. Butte et al. [20] investigated the association between ST and change in BF% and found that objectively measured ST at 3–5 years was not associated with a change in BF% at 1-year follow-up after adjusting for several child and maternal characteristics and household factors [20]. Similarly, another longitudinal study found no relations of objectively registered ST at 4.5 years with fat mass index and BF% a year later after adjusting for gender, age, AWT, and VPA [19]. These results align with the lack of associations between ST and BF% in the current study before controlling for confounders. However, after adjustment, the associations between ST in preschool and BF% in school became positive among girls. Partially in line with the current study’s results among girls, inactive children aged 3–5 years were 3.8 times more likely than active peers to have an increasing triceps skinfold incline gradient before starting first grade after controlling for age, TV viewing, energy consumption, triceps skinfold thickness, parental BMI, perceived competence, and social acceptance score [13]. Also, in active girls, skinfold thickness increased by 1 mm during the period from preschool to school, while the skinfold thickness among inactive girls increased by 1.75 mm. The skinfold thickness among active boys decreased by 0.75 mm but increased by 0.25 mm among inactive boys [13]. Earlier cross-sectional studies among Estonian schoolchildren at 7–9 years [55] and 10–12 years [56] have also found an association between ST and BF% comparable to that found in the present study among Estonian preschool-aged girls.

It is proposed that PA, the largest modifiable component of energy expenditure, may assist in attaining a healthy weight by encouraging energy balance [16]. The sparse number of studies on objectively measured MVPA and body fat indices among preschoolers report contradictory results [15,16,17,18,19,20,21,22]. Collings et al. [16] specifically emphasized the importance of VPA (3368 counts per minute in their work), since MPA was unrelated to body fatness and MVPA gave an inverse association with body fatness due to VPA among 4-year-old children [16]. In a later study, Collings et al. [17] reported that more than 3500 counts per minute (corresponding to the high end of MPA and VPA in our study) among children aged 11 months–5 years was negatively related to the sum of skinfolds after adjustment for age, gender, ethnicity, deprivation in an area, AWT, and season. The strength of the relationship was greater with higher PA intensity, peaking for activity with more than 6000 counts/min [17]. Despite that, no associations of different PA intensity zones and total PA with the sum of skinfolds, BMI, or waist circumference were found in a group of children aged 11 months to 5 years [17]. Corresponding to the missing associations between MVPA and BF% in unadjusted and adjusted analyses in the present study, Bürgi et al. [22] identified that total PA, MPA, and VPA were not associated with BF% cross-sectionally among 4–6-year-old children after adjustment for sociocultural factors. Also, no longitudinal relationship was found between baseline PA and changes in BF% during the 9-month follow-up [22]. While higher MVPA was associated with lower BF% at 4.5 years after controlling for gender, age, AWT, ST, maternal and paternal BMI, and educational level [18], those associations disappeared longitudinally [19]. Additionally, Butte et al. [20] obtained concurrent results in a cross-sectional and longitudinal study among preschool-aged children. Unlike the current study, Basterfield et al. [15] demonstrated gender-specific associations among schoolchildren at 7–9 years—a reduction in MVPA was linked to an increase in fat mass index only among boys [15]. Additionally, a study among children aged 9 to 10 years, originating from different countries, including Estonia, demonstrated that, after controlling for gender, birth weight, study place, puberty, and parents’ BMI, MVPA had a significant but weak relationship with adiposity, explaining <1% of the variation [57].

There are several possible reasons for the discrepancies observed in the results of previous studies. First, investigators have applied different body fatness measurements such as skinfold thickness [13,17,21], bioelectric impedance [15,22], dual-energy X-ray absorptiometry [16,20], air-displacement plethysmography [18,19], BMI [14,15,21], and waist circumference [14,17,21]. Second, study design (cross-sectional vs. longitudinal) might affect the associations found in studies. Third, the findings are influenced by the confounders applied. To account for baseline differences in the outcome of interest, we adjusted for the baseline value of the outcome, as did Bürgi et al. [22], Basterfield et al. [15], Moore et al. [13], and Haapala et al. [48], while the other longitudinal studies did not [19,20,21]. Additionally, unlike the current study, others have adjusted for birth weight [16], sleep duration [16], height [16,20], energy intake [13], race/ethnicity [14,16,20], weekly daycare hours [20], TV viewing [13], perceived competence and social acceptance score [13], socioeconomic deprivation [15,17], season of assessment [17], VPA or MVPA in the models with ST and vice versa [15,16,19,20,21], parental migrant status [22], smoking during pregnancy [16], maternal age [20], duration of breastfeeding [16], maternal BMI [16,18,20], paternal BMI [18] or parents’ BMI [13], maternal [16,18,20] and paternal education [18], family income, and size [20]. Most studies also controlled for gender and did not study boys and girls separately [15,16,17,18,19,20,21,22].

The second goal of our study was to explore whether CRF and MF at preschool age are related to BF% in the first school year among boys and girls. The second main finding was that higher MF in preschool was related to lower body fatness in the first school year among girls but not in boys when adjusted for potential confounding variables. More precisely, while the unadjusted analysis revealed that higher MF in preschool was related to lower body fatness in school both among boys and girls, those associations remained significant only among girls after controlling for age, BF%, and parental education at baseline.

Only a few cross-sectional [23,24,28] and longitudinal studies [25,26] have investigated the associations of CRF and upper- and lower-limb muscular strength with body fatness among preschool children. Correspondingly to the current study, Latorre Román et al. [24] found that CRF was unrelated to BMI after adjustment for age among children at 3–6 years. They also found that SLJ test results were not associated with BMI [24]. On the contrary, in another cross-sectional study among preschoolers aged 4.5 years, CRF and lower-limb muscular strength were inversely associated with BF% and fat mass index once adjusted for age, gender, fat mass index, and fat-free mass index. At the same time, upper-limb muscular strength was unrelated to body fatness parameters [23]. Those findings were confirmed by a longitudinal study among children aged 4.5 years with a 1-year follow-up [25]. Likewise, in another longitudinal study among preschool children aged 6.6 years, higher CRF and relative lower-limb muscular strength were associated with lower BF% and fat mass index a year later, when children had entered first grade in school, once adjusted for gender and age, maternal BMI, and educational attainment. Additionally, higher relative upper- and lower-limb muscular strength were associated with a lower waist-to-height ratio. Mixed results have been reported for the relationships among relative upper-limb muscular strength, BF%, and fat mass index [26]. Among primary school children aged 6 years, CRF, upper-limb muscular strength (flexed arm hanging), and lower-limb muscular strength predicted decreased body fat growth over the following 9 years after controlling for gender [58].

While the previously described studies did not identify associations specifically among boys and girls, Agha-Alinejad et al. [28] studied 5–6-year-old boys and girls separately. The authors reported that upper-limb muscular strength (modified pull-ups) was inversely correlated with most adiposity parameters including BF% in both genders. This corresponds to our results in boys and girls regarding the inverse association between MF and BF% from unadjusted analysis. As Agha-Alinejad et al. [28] adjusted only for weight and did not precisely report those results, we cannot compare the results from the adjusted analysis. Also, higher CRF correlated with lower BF% among boys, but not among girls [28]. This is in agreement with the inverse relation between CRF and BF% (β = −0.287 *p* = 0.032) among preschool-aged boys in the present sample of children, as well as with missing relations among girls (data not shown). However, our longitudinal findings indicate that CRF in preschool is unrelated to BF% in school among both genders. Again, there are many reasons for nonuniformities in the results between studies, including regression adjustment and body fatness measurement, which was assessed by skinfolds in the present and some other [26,27,28,58] studies, air-displacement plethysmography in some studies [23,25], and BMI [24,26,27] in other studies. There were also some differences in tests applied to measure children’s PF. While most investigators used the 20 m shuttle run test to measure participants’ CRF, one study applied half-mile time [27]; a hand dynamometer was most widely used to test upper-limb muscular strength, yet flexed arm hang [58] and modified pull-ups [28] were also used. Finally, unlike comparative research, the mean of the relative upper- and lower-limb muscular strength z-score was used in our study to represent muscular fitness [47].

The strengths of this study are the objective monitoring of PA and the application of standardized physical fitness testing in a longitudinal design. However, we also present some weaknesses of the current study. The first weakness is the low number of participants. Second, the thickness of skinfolds was measured to determine body fatness. Third, there were some accelerometer-related limitations, such as the inability to assess water-based (e.g., swimming) and other non-ambulatory activities (e.g., cycling) [59,60]; U-shaped reactivity, especially in young children [61]; and the hip-mounted accelerometer underestimating sedentary behaviours and MVPA and overestimating LPA and breaks in sedentary activities [62]. All of these can lead to bias in the objective PA and ST estimates. Fourth, we cannot exclude residual confounding. Fifth, due to the observational nature of this study, we were unable to establish causality.

## 5. Conclusions

In conclusion, lower ST and higher muscular fitness at preschool age are related to lower body fatness in first grade among girls, but not among boys. MVPA and CRF at preschool age are not related to body fatness in school in boys and girls.

## Figures and Tables

**Table 1:** Baseline data.

Variable	Boys (*n* = 40)	Girls (*n* = 37)	*p*
Age (years) ^1^	7 (1)	6 (1)	0.240
Height (cm)	127 (5.5)	124 (6.6)	**<0.001**
Weight (kg) ^1^	26 (7)	24 (6)	**0.010**
BMI (kg/m^2^) ^1^	16.1 (2.5)	15.5 (2)	0.323
Children with overweight and obesity (%)	17.9	9.9	0.170
BF%	20.6 (4.6)	22.6 (5)	**<0.001**
Physical activity			
AWT (min/day) ^1^	774 (93.1)	752 (96)	0.151
ST (min/day) ^1^	386 (74.9)	356 (76.4)	0.237
MVPA (min/day) ^1^	62.9 (28.9)	58.5 (28.3)	**0.003**
Physical fitness test			
20 m shuttle run (laps) ^1^	18 (19)	18 (10)	0.383
Handgrip strength (kg) ^1^	11.5 (3)	9.9 (2.7)	**0.007**
Handgrip strength/weight (kg/kg)	0.4 (0.1)	0.4 (0.1)	0.299
Standing long jump (cm)	122 (19)	117 (15.6)	0.061
Muscular fitness z-score	−0.1 (0.6)	−0.05 (0.5)	0.797

Data are from Student’s *t*-test or the Mann–Whitney U-test, or the chi-square test for categorical variables, and are presented as means (standard deviations), medians (interquartile ranges ^1^), or percentages (%). BMI—body mass index; BF%—body fat percentage; AWT—accelerometer wear time; ST—sedentary time; MVPA—moderate-to-vigorous physical activity. Bold values denote *p* < 0.05.

**Table 2:** Multiple regression analysis demonstrating the associations of physical activity, sedentary time, and physical fitness in preschool with body fatness in school.

	BF% in School			
	Boys		Girls	
Variables in Preschool	β	*p*	β	*p*
Unadjusted (*n* = 48 boys, 40 girls)				
MVPA	0.054	0.702	−0.174	0.254
Adjusted (*n* = 48 boys, 40 girls)				
MVPA	0.094	0.365	−0.160	0.101
Age	0.118	0.232	0.129	0.193
BF%	0.791	**<0.001**	0.808	**<0.001**
Education	−0.004	0.968	−0.117	0.216
AWT	−0.052	0.634	−0.081	0.413
Unadjusted (*n* = 48 boys, 40 girls)				
ST	−0.189	0.180	−0.008	0.960
Adjusted (*n* = 48 boys, 40 girls)				
ST	−0.302	0.058	0.587	**0.021**
Age	0.153	0.118	0.128	0.175
BF%	0.771	**<0.001**	0.833	**<0.001**
Education	−0.001	0.990	−0.073	0.436
AWT	0.218	0.161	−0.578	**0.021**
Unadjusted (*n* = 56 boys, 54 girls)				
CRF	−0.248	0.058	−0.098	0.456
Adjusted (*n* = 56 boys, 54 girls)				
CRF	−0.059	0.496	−0.014	0.875
Age	0.186	**0.027**	0.144	0.100
BF%	0.766	**<0.001**	0.828	**<0.001**
Education	−0.048	0.591	−0.053	0.558
Unadjusted (*n* = 58 boys, 55 girls)				
Muscular fitness	−0.263	**0.042**	−0.349	**0.006**
Adjusted (*n* = 58 boys, 55 girls)				
Muscular fitness	0.032	0.716	−0.231	**0.009**
Age	0.139	0.100	0.222	**0.011**
BF%	0.818	**<0.001**	0.789	**<0.001**
Education	−0.006	0.944	−0.054	0.497

BF%—body fat percentage; β—standardized regression coefficient; MVPA—moderate-to-vigorous physical activity; AWT—accelerometer wear time; ST—sedentary time; CRF—cardiorespiratory fitness. Bold values denote *p* < 0.05.

## Data Availability

The datasets used in this study are available from the corresponding author upon reasonable request due to privacy and ethical reasons.
